# Electromyographic analysis of selected shoulder muscles during a rugby football tackle

**DOI:** 10.1186/1758-2555-1-10

**Published:** 2009-05-21

**Authors:** Lee Herrington, Ian Horsley

**Affiliations:** 1 Senior Lecturer in Sports Rehabilitation, Directorate of Sport, University of Salford, UK; 2 Head Physiotherapist, Back in Action Physiotherapy, Wakefield, UK

## Abstract

**Background:**

Epidemiological studies have shown that the incidence of shoulder injuries is increasing in rugby and the majority are related to the contact/tackle phase of play. However, no data currently exists that describes preparatory muscle activity during tackle. This information could aid in guiding training and rehabilitation, if available. The purpose of the study was to assess the sequence of onset of EMG activity of selected scapulohumeral muscles during rugby tackle. 15 healthy professional rugby players participated in the study. Surface EMG activity was assessed for timing of onset relative to time of impact during a modified tackle activity in pectorialis major, biceps brachii, latissimus dorsi, serratus anterior and infraspinatus muscles.

**Results:**

Onset of activity occurred in all muscles prior to impact. Factorial ANOVA showed significant differences between muscles in activation timing (p = 0.0001), paired t-tests revealed that serratus anterior was activated prior to all other muscles tested (p < 0.04, for all comparisons), with comparison between all other muscles showing no significant differences (p > 0.05), except pectorialis major on all comparisons showed significantly later activation timing than all other muscles (p < 0.001).

**Conclusion:**

Muscle activation timing may if not properly balanced around the shoulder girdle expose the glenohumeral joint to excessive load and stress. This paper demonstrates a simple method which sets out some preliminary normative data in healthy players. Further studies relating these data to injured players are required.

## Background

Several authors have highlighted that shoulder injuries are becoming more severe within professional rugby [1,2] and tackling or being tackled being responsible for a majority of these reported shoulder injuries [1-3]. Despite the weight of evidence linking tackling within rugby to shoulder injury, there are no studies with reporting muscle activity around the shoulder girdle during the tackle within rugby football.

Electromyography (EMG) has been utilised as a tool for analysing the function of muscles for a number of decades, in both normal and injured subjects. Several authors have analysed muscle recruitment activity around the lumbar spine and abdomen in patients with and without low back pain [4], cervical muscle function [5] knee and patellofemoral joint [6,7] and there are a few studies related to the shoulder girdle [8,9]. In many sports precise motor acquisition and rapid reaction time are important in preventing injury to the joint. An altered interaction between the dynamic and passive stabilizers may predispose a sportsman to an increased incidence of joint disruption [10]. Increased muscle stiffness is likely to augment joint stiffness and so enhance the functional stability of the joint [11]. Both direct contraction of agonist and co contraction of antagonist muscles groups have been shown to increase joint stiffness [12], it would follow then that appropriate (and early) activation of muscles is likely to increase joint stability.

Altered dynamic control (muscle contraction) around the shoulder complex has been shown to be a significant factor in shoulder dysfunction [9]. The balance of muscle force couples around the shoulder complex has been shown to be more important than muscle strength to establish normal joint function [13]. The role of proprioception in allowing a feedback mechanism to work, which in turn allows a synergistic contraction of muscle groups, may be vital both for normal functioning of the muscle groups of the shoulder joint and in protecting the shoulder against potential instability [14]. Coactivation of the dynamic stabilizing force couples around the glenohumeral joint is necessary to afford joint stability with active movement by producing joint compression and maximal joint congruency [14], and thus preventing excessive humeral head translation on the glenoid.

During the tackle, the shoulder is part of a kinetic chain of energy, in which the body is considered as a linked system of articulated segments [15]. The force is transmitted through the kinetic chain, form the legs, hips and trunk, to the shoulder girdle at the point of impact within the tackle, whereby rapidly developing deceleration forces will be developed within the shoulder girdle that should be attenuated by a coordinated recruitment of the muscles.

The purpose of this study is to define the sequence of muscular activation patterns in selected shoulder girdle muscles during a "front on" tackle in an asymptomatic

## Methods

### Subjects

Following Ethical approval by the University of Sheffield 15 full time professional rugby union players (mean age 22+/-1.4 years range 19–35) were recruited to participate in the study after informed consent was taken. All these individuals had no history of injury to the shoulder, cervical or thoracic spine in the previous 12 months.

### Electrode placement

**Figure 1:**
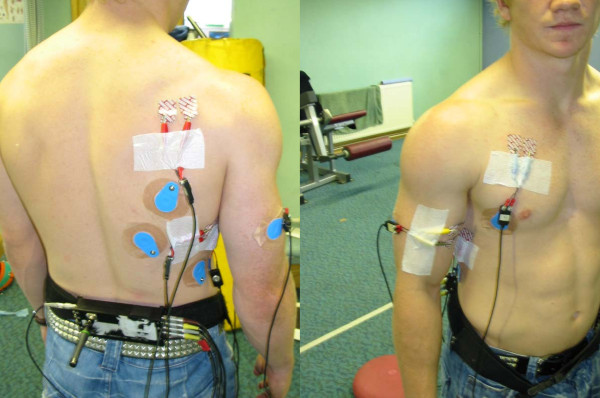
**Electrode Placement**.

Electrodes were placed in line with the recommendations of Cram and Kashman [16]. The electrodes were placed at specific sites where the muscle was superficial and the electrodes were placed parallel to the muscle fibres, in the mid-line of the muscle belly. The muscles which were selected were the ones which allowed for easy access for surface EMG (sEMG), and which have been regarded as responsible for stabilization (serratus anterior, infraspinatus and biceps) or mobilization (pectoralis major and latissimus dorsi) of the shoulder complex (see figure 1). Although the upper fibres of trapezius were accessible, it was decided not to evaluate its activity, as it is also recruited in maintaining the cervical spine position and the alteration in head and neck position during tackling would potential have an effect on the sEMG activity which was recorded at the shoulder during the tackle.

#### Serratus Anterior

Two active electrodes were placed 1 cm apart, just below the axillary area, at the level of the inferior angle of the scapula, just medial to the latissimus dorsi. Correct electrode placement was carried out by noting sEMG activity during resisted protraction of the arm at 90 degrees flexion.

#### Biceps Brachii

Two active electrodes were placed 1 cm apart, and 3 cm above the myotendinous junction. Correct electrode placement was carried out by noting the sEMG activity during resisted elbow flexion with the elbow flexed to 90°.

#### Infraspinatus

Following identification of the spine of the scapula, two electrodes were placed 1 cm apart parallel to and approximately 4 cm below the scapular spine on the lateral aspect of the infraspinous fossa. Correct electrode placement was carried out by noting the sEMG activity during resisted lateral rotation of the arm whilst at 90 degrees abduction and with 90 degrees elbow flexion.

#### Pectoralis Major

(Clavicular fibres). Two active electrodes were placed 2 cm below the clavicle and medial to te axillary fold at an oblique angle 1 cm apart. Correct electrode placement was confirmed by noting the sEMG signal during resisted humeral adduction at 90 degrees of forward flexion.

#### Latissimus Dorsi

Two active electrodes were placed 1 cm apart, approximately 4 cm distal to the inferior angle of the scapula, at an oblique angle of approximately 25 degrees. Correct electrode placement was confirmed by noting sEMG signal activity during resisted humeral extension from 120 degrees forward flexion.

### Electromyography technique

Simultaneous recordings of the sEMG activity from the Pectorialis Major, Biceps Brachii, Latissimus Dorsi, Serratus Anterior and Infraspinatus muscles were made during the procedures outlined below. Prior to mounting the recording electrodes, the skin surface was prepared by light abrasion (Nuprep, SLE Ltd) and cleaning with alcohol swabs. Two silver/silver chloride bipolar electrodes (Medicotest UK, type N10A), with a 1 cm inter-electrode distance (centre to centre) were placed midline on one of the prepared muscle site locations outlined below. A ground electrode (Medicotest, UK, type Q10A), was placed at an electrical neutral site; the sternum. The sEMG was high and low pass filtered between 10 and 500 Hz respectively (Neurolog filters NL 144 and NL 134, Digitimer, UK), preamplified (×1000), (Neurolog remote AC preamplifier NL 824, Digitimer, UK), amplified (×2) (Neurolog isolation amplifier, NL 820, Digitimer, UK) and A/D converted at a rate of 2000 Hz (KPCI 3101, Keithley instruments, UK). To determine the sEMG signal on/off, a computer aided algorithm was used (Testpoint, Keithley instruments, UK) to allow a threshold value to be calculated from 3 standard deviations above baseline [11]. To ensure the validity of the computer derived sEMG onsets each trace was also visually inspected in order to ensure that movement artefact or other interference was not incorrectly identified as a muscle onset [17]. The impact of the tackle was determined from a pressure change detected in a pressure switch placed on the anterior aspect of the shoulder and visual inspection of the sEMG traces.

### Procedure

**Figure 2:**
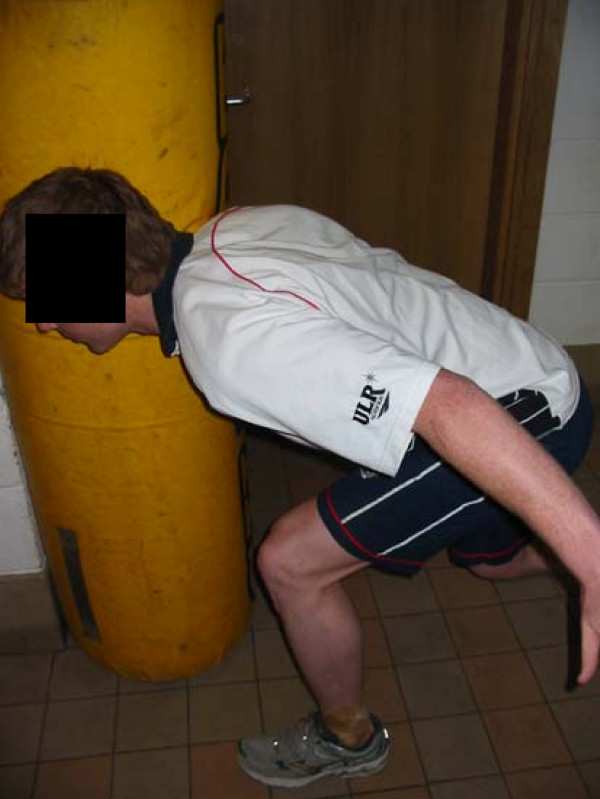
**Foot and body position at contact**.

Each subject aligned the contra-lateral foot to the tackling shoulder alongside the tackle bag, the trunk was flexed to approximately a 90° angle between the trunk and thigh, knees flexed to 45° and shoulder abducted to about 60° (figure 2), this was the "set" position. Upon a command from the investigator, the subject prepared on the word "set" and then on the command "hit", the player pushed forwards through the legs, extending at the hips and knees (but keeping their feet in place) and hit the tackle bag with the chosen shoulder (Figure 2). The sEMG data was recorded from the command "set" until contact was made with the tackle bag. This was repeated 5 times for each shoulder, with the average data being calculated and used for analysis.

### Analysis

Data were analysed using the statistical software package SPSS (version 12). Differences in time of onset between muscles were analysed with a factorial ANOVA with two factors (side (left or right) and muscle). The critical alpha level chosen α = 0.05 for all analysis. Paired t-tests were used to evaluate specific differences found (corrected for family-wise inflation of type 1 error with Bonferroni corrections). In order to assess the test- retest reliability of the muscle onset timing, the second and the fifth repetition for each subject for all muscles was compared using intra-class correlation coefficient (ICC) to assess both the degree of correspondence and agreement between the tests [20]. These results are displayed in table 1. Measurement variability was calculated using 95% confidence limits (CI) using the formula [18].

**Table 1:** Test-retest reliability of the muscle onset times

	Pectoralis Major (Msec)	Biceps Brachii (Msec)	Latissimus Dorsi (Msec)	Serratus Anterior (Msec)	Infraspinatus (Msec)
Mean differenc	1.7	1.3	1.3	1.9	2.0

Standard Deviation (SD)	1	1	0.6	1.1	1.1

Standard error of measurement (SEM)	0.33	0.39	0.22	0.35	0.4

Confidence interval (95%)	1.06–2.34	0.87–2.06	0.87–1.73	1.21–2.59	1.22–2.78

ICC_3, k_	0.89*	0.85*	0.87*	0.9*	0.87*

* Statistical Significant (p < 0.01)

95% CI = 1.96 × SEM

SEM = SD × √1-ICC [18].

## Results

**Figure 3:**
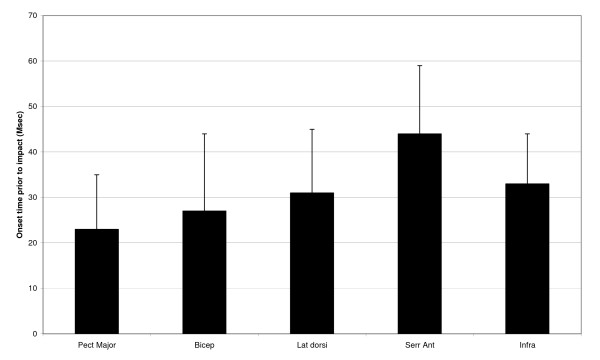
**Mean onset time prior to impact (MSec) for each muscle**. Muscle: Pect Major: Pectoralis Major. Bicep: Biceps Brachii. Lat Dorsi: Latissimus Dorsi. Serr Ant: Serratus Anterior. Infra: Infraspinatus.

The results of the study are shown in figure 3 and table 2. Figure 3 and table 2 show the timing differences of the muscles relative to the tackle impact, the larger the time, the earlier the muscle contracted prior to impact.

**Table 2:** Mean onset times prior to impact (MSec) for each muscle

Muscle	Mean Onset time	Confidence interval (95%)
Pectoralis Major	20.7	16.3–25.1

Biceps Brachii	27	23–31

Latissimus Dorsi	37.8	35–40.6

Serratus Anterior	41.2	38.2–44.2

Infraspinatus	35.4	30.6–40.2

Factorial ANOVA revealed a significant effect for activation timing between muscles (p < 0.0001) and a significant interaction between muscle and limb (p = 0.023) but the main effect of limb (side) on activation timing was not a significant one (p = 0.16). These results would appear to indicate that activation timing differs between muscles, but not between sides. Paired t-tests (with Bonferroni corrections) revealed that serratus anterior was activated prior to all other muscles tested (p < 0.04, for all comparisons), with pair-wise comparison between biceps, latissimus dorsi and infraspinatus muscles showing no significant differences (p > 0.05) in timing and pectorialis major on all comparisons showing significantly later activation timing than all other muscles (p < 0.001).

## Discussion

The rational for using sEMG to study muscle activation during a standardized rugby tackle is to provide a better understanding of muscle firing patterns during this sport-specific movement. By understanding the muscle activity during the tackle, the sports medicine practitioner will be able to provide rugby players with the most effective training method for optimal muscle-specific conditioning. Furthermore, if the sequence of muscle activation around the shoulder during the tackle is known, then a more specific rehabilitation programme can be developed, which may facilitate a quicker and safer return to competition following injury. The results of the study showed that the onset timings of the muscles were consistent (r = 0.85–0.9) with only a small variability between repetitions and no significant difference in timing between sides (p = 0.16). The findings indicate a consistently earlier activation of serratus anterior muscle prior to impact, ahead of all the other muscles. pectoralis major was activated later than all the other muscles, but was still recruited prior to impact.

Altered dynamic control of muscles around the shoulder complex has been shown to be a significant factor to shoulder dysfunction [9], with the balance of muscle force couples around the shoulder complex has been shown to be a more important factor than muscle strength in the re-establishment of normal joint function [19]. Furthermore, studies on subjects with unstable shoulders have shown widely differing patterns of muscle activation onset, with failure of the rotator cuff and biceps to be activated prior to pectorals at the onset of movement. The altered muscle recruitment will disturb normal scapulohumeral rhythm and potentially cause inappropriate positioning between the humeral head and the glenoid, which may result in subsequent injury [8]. Kibler [19] described the mechanism whereby as the humeral head moves on the glenoid, the scapula rotates simultaneously, thereby maintaining the correct relative positions, which will be responsible for providing the optimal length-tension relationship of the rotator cuff, this requires considerable dynamic muscular control, uncontrolled motion could lead to the overloading of some tissues within the shoulder and pathology. The tackle in rugby has been linked to the aetiology of shoulder injuries within the sport [1] yet little is know of the mechanics of tackling, this paper provides an insight into the organisation of muscle action within a controlled tackle. As with the studies highlighted above deviation from this normal pattern of recruitment may result in pathology and would be the source of future research hypothesis. However, it must be noted that a rugby tackle may often occur in uncontrolled situations such as side to side tackle, indirect tackle during a fall, combinations of pull, push or direct impact etc. Further studies are needed to identify which of these mechanisms are most likely cause shoulder injuries in professional rugby.

Previous authors have demonstrated that preparatory hamstring muscle activity within the knees of ACL deficient patients, produces muscle stiffness which then increases muscle spindle sensitivity and reduces EMD [21] potentially controlling unwanted tibial translation at the knee. Solomonow et al. [20] have demonstrated the existence of a spinal reflex between the shoulder capsule and the shoulder muscles within the feline model, which may modulate activity in a similar manner. This reflex has also been demonstrated within the human shoulder by Jerosch et al. [22], but they postulated that this reflex was too slow to provide joint stabilization. The early activity seen in this study of serratus anterior may be an example of preactivation/feedforward within the tackle situation, thus providing a rapid compensation in response to external forces, and hence providing glenohumeral joint stability indirectly by stabilising the scapula.

Research literature has identified serratus anterior as one of the primary muscles for maintaining scapluohumeral rhythm [8], with lack of recruitment reducing scapular lateral rotation and protraction, allowing the humeral head to translate anteriorly and superiorly [23]. Due to this stability role, late, or reduced activation of serratus anterior could reduce the ability of the shoulder girdle to resist the high deceleration forces experienced at the point of impact within the tackle. Within subjects who demonstrated anterior instability of the glenohumeral joint, Glousman et al. [24] reported that there was increased activity of the long head of biceps during throwing, suggesting that it helped compensate for any anterior instability present. Thus in pathological shoulders we would expect to see an alteration in timing of both serratus anterior and, possibly, long head of biceps.

Further research needs to be carried out on players with different shoulder injuries to analyze whether these shoulders show an altered muscle activation pattern in comparison to their asymptomatic shoulders. If these patterns were identified then this information could be used to help design upper limb, functional training programmes to prepare players for rugby, and help us to evaluate in particular late rehabilitation after shoulder injuries and surgery before returning to full play.

There were several limitations of the study. The first being, that due to the nature of sEMG, we were limited to the muscles which were easily accessible to record activity. This prevented us from being able to analyse other muscles, such as subscapularis, and teres minor. Secondly, this was a lab-based study and the position of the arm was set in a standard position for all subjects. This may not have been the preferred arm position for all individuals studied (90 degrees abduction). Also the tackle bag was stationary and of uniform shape and density, unlike an opposition rugby player. Furthermore, the tackler was tackling from one pace away and the tackle bag was stationary, thus reducing the momentum within the system which may have an effect on the muscle recruitment.

Another factor not experienced in this test position is any angular rotation. Within a game situation, during the tackle, the attacker would be taking action to avoid the tackler, and thus in contact there would be an element of rotation upon contact, which is not produced in this test situation. Finally, during the test, there is a relatively long preparatory phase for the tackle, with the subject being in a comfortable set position prior to the tackle being executed. This would not be the case within rugby. There would be a much shorter preparatory time, generally, and the tackler would generally be moving forward. These limitations could possibly be addressed by repeating the study with the subject carrying out a tackle on an oncoming opponent, although a study of this nature may have severe methodological limitations.

## Conclusion

Muscle activation timing may if not properly balanced around the shoulder girdle expose the glenohumeral joint to excessive load and stress. This paper demonstrates a simple method which sets out some preliminary normative data for muscle activation patterns during rugby tackle in healthy players. Further studies relating these data to injured players are required.

## Competing interests

The authors declare that they have no competing interests.

## Authors' contributions

Both authors participated fully and equally in all areas of conceptual design, data collection & analysis, and dissemination.
